# The Effect of (Mg, Zn)_12_Ce Phase Content on the Microstructure and the Mechanical Properties of Mg–Zn–Ce–Zr Alloy

**DOI:** 10.3390/ma15134420

**Published:** 2022-06-22

**Authors:** Yuguang Li, Feng Guo, Huisheng Cai, Yiwei Wang, Liang Liu

**Affiliations:** School of Material Science and Engineering, Inner Mongolia University of Technology, Hohhot 010051, China; liyuguang0401@163.com (Y.L.); jingxing_chs@163.com (H.C.); wangyiweics@163.com (Y.W.); nmggydx2022@126.com (L.L.)

**Keywords:** Mg–Zn–Ce–Zr alloy, (Mg, Zn)_12_Ce phase, phase separation, thermal deformation, quantitative analysis

## Abstract

The quantitative study of rare earth compounds is important for the improvement of existing magnesium alloy systems and the design of new magnesium alloys. In this paper, the effective separation of matrix and compound in Mg–Zn–Ce–Zr alloy was achieved by a low-temperature chemical phase separation technique. The mass fraction of the (Mg, Zn)_12_Ce compound was determined and the effect of the (Mg, Zn)_12_Ce phase content on the heat deformation organization and properties was investigated. The results show that the Mg–Zn–Ce compound in both the as-cast and the homogeneous alloys is (Mg, Zn)_12_Ce. (Mg, Zn)_12_Ce phase formation depends on the content and the ratio of Zn and Ce elements in the initial residual melt of the eutectic reaction. The Zn/Ce mass ratios below 2.5 give the highest compound contents for different Zn contents, 5.262 wt.% and 7.040 wt.%, respectively. The increase in the amount of the (Mg, Zn)_12_Ce phase can significantly reduce the critical conditions for dynamic recrystallization formation. Both the critical strain and the stress decrease with increasing rare earth content. The reduction of the critical conditions and the particle-promoted nucleation mechanism work together to increase the amount of dynamic recrystallization. In addition, it was found that alloys with 6 wt.% Zn elements tend to undergo a dynamic recrystallization softening mechanism, while alloys with 3 wt.% Zn elements tend to undergo a dynamic reversion softening mechanism.

## 1. Introduction

Magnesium alloys have become one of the most promising lightweight structural materials today due to their low density, high specific strength, and specific stiffness [1,2,3], and they are used in a large number of applications in the aerospace, military, and electronics industries [4]. In addition, magnesium is widely used in medicine for medical implant applications, one of which is to be a fixation for the acetabular cup component of total hip prostheses [5]. In recent years, researchers have developed a large number of new high-strength magnesium alloys by designing material preparation methods [6,7,8,9] and introducing various rare earth elements. Mg–Zn–Zr series magnesium alloys can be applied to a variety of high-load, high-yield parts and large complex forgings because of their high strength, good plasticity, corrosion resistance, and good heat treatment and processing properties. The application of rare earth elements (RE) in Mg–Zn–Zr series magnesium alloys such as Ce, Y, Gd, Tb, Dy, Ho, or Er [10] further enhances the performance of this series of alloys and compensates for the poor thermal stability performance of Mg–Zn–Zr series alloys, which is one of the hot areas of current research.

The addition of Ce is expected to improve the strength and the heat resistance of Mg–Zn–Zr alloys due to its huge reserves in the earth’s crust and easy extraction, especially in the Mg–Zn–Zr system. According to the Mg–Ce binary phase diagram [11], the solid solution of element Ce in the magnesium matrix is extremely low, so the improvement of the properties of the Mg–Zn–Ce–Zr alloy acts mainly through intermetallic compounds. Currently, thermodynamic analysis of Mg–Zn–Ce–Zr alloys (Mg-rich angular region) gives information on the formation temperature, transformation reaction equations, and structure of various compounds [12,13]. The addition of Ce improves the yield strength (YS), ultimate tensile strength (UTS), and ductility of the alloy by refining the grains [14], promoting the formation of dynamic recrystallization, forming high density precipitation, and weakening the weave [15]. The mixed addition of Ce and other elements (Y, Ca, etc.) also contributes to the strength and ductility of the alloy [11,16,17,18]. The above reports indicate that rare earth compounds such as (Mg_1−x_Zn_x_)_11_Ce and (Mg_1−x_Zn_x_)_12_Ce are the key to playing a strengthening role. The organizational characteristics of Mg–Zn–Ce compounds, including morphology, distribution, and quantity, all influence their role in the alloy, among which the quantity of Mg–Zn–Ce compounds has been less studied. The quantitative study of the effect of the quantity of rare earth compounds on strengthening effect has become one of the important tasks of current research.

Several methods have been used for the analysis of the content of rare earth compounds, among which the older point parameter method is more limited. Atomic probe chromatography (APT) [19,20], although the most accurate, is not widely available with expensive equipment. The phase separation method is a more widely used analytical method, and it has some applications in Mg-Al-Zn magnesium alloys [21], but the number of rare earth compounds studied by the phase separation technique in Mg–Zn–Ce–Zr magnesium alloys is still a gap.

In summary, the quantitative study of the (Mg, Zn)_12_Ce phase and its content regulation method are the key elements in the study of Mg–Zn–Ce–Zr alloys. To this end, a novel chemical phase separation method was designed to investigate the mass fraction of the (Mg, Zn)_12_Ce phase in different composition alloys. In the Mg–Zn–Ce–Zr alloy, the Zn element can form the Zn–Zr phase before the eutectic reaction at the early stage of solidification, and it may form the Mg–Zn phase during the eutectic reaction. Therefore, in this study, it is assumed that all Zr forms Zn–Zr compounds, and the content of Zn and Ce in the solidified melt can be obtained by using the difference between the total amount of elements and the measured solid solution amount. Further, the effect of Zn and Ce content on the amount of Mg–Zn–Ce ternary compound formation is discussed in order to provide help for the design and the improvement of new high-strength alloys.

## 2. Materials and Methods

The Ce elements were added to the Mg–Zn–Zr alloy to form the alloy used for the experiments. The added Ce content was 0.5 wt.%, 1.0 wt.%, and 1.5 wt.%, respectively, and the Zn content was 3 wt.% and 6 wt.%. Pure magnesium and intermediate alloys (Mg–Zn, Mg–Ce and Mg–Zr) were melted under slag protection to prepare semi-continuous ingots of 210 mm. The actual chemical composition test results of the ingots are shown in Table 1. The experimental specimens were intercepted at 1/2 the same height as the ingot, with a diameter of 12 mm. The samples were sealed in quartz tubes filled with argon gas for homogenization heat treatment. The homogenization treatment was carried out in a chamber resistance furnace at a temperature increase rate of 10 °C/min to 475 °C, held for 12 h, and then immediately water-cooled. The specimens were processed into short bars of 10 mm, 15 mm. Thermal deformation experiments were conducted using a Gleeble-3500 thermal simulation tester with a heating rate of 3.3 °C/s to 400 °C, a holding time of 3 min, a strain rate of 0.1 s^−1^, and a deformation of 50%. The deformed specimens were water-cooled immediately after deformation and used for deformation tissue observation.

The low-temperature phase separation of the alloy was performed by chemical methods. The solid solution phase in the alloy reacts with the solution and then decomposes and dissolves into the solution, while the compound phase remains in the solution in its original form. The phase separation solution uses methanol as the solvent, ammonium benzoate as the surface passivator of the compound, 2,2′-bipyridine as the complexing agent of Zn ions, salicylic acid as the inhibitor of hydrolysis products, and dioxane as the chemical corrosion inhibitor of the magnesium alloy. The reaction between the alloy and the separation solution was carried out at −20 °C with a reaction time of 2 h. After the reaction, the solid–liquid was separated by the centrifugation + membrane method. The liquid phase was used to determine the content of rare earths and Zn elements in the solid solution, and the solid phase was used for the physical identification and the morphological observation of the compounds.

**Figure 1:**
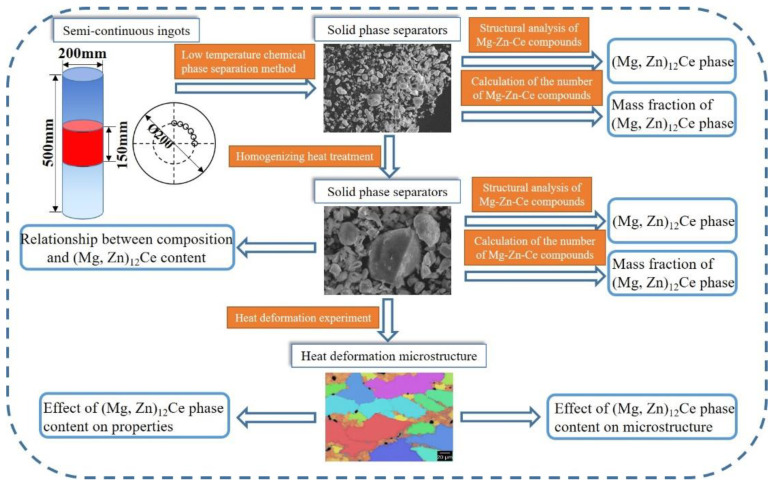
SEM microstructure images of the as-cast alloys.

The HITACHI SU8220 scanning electron microscope (SEM) was used to observe the morphology of the metallographic sample and the separated solid phase of the alloy. The composition of the micro-area was analyzed by an energy dispersive spectrometer (EDS). The composition of the as-cast alloy and the solid phase separation was analyzed by D/Max 2500/PC X-ray diffraction (XRD). The X-ray diffractometer had a Cu-k target, and its working voltage was 40 kV and its current was 300 mA. The scanning speed was 1 degree per minute, and the scanning step was 0.02 degrees. The scanning angle range was 20–80 degrees. The transmission electron microscope (TEM) analysis sample of the as-cast experimental alloy was prepared by the ion thinning method. The phase morphology observation and the selected area diffraction analysis were carried out by the FEI Talos F200X transmission electron microscope. The electron acceleration voltage was 200 kV. Figure 1 shows the workflow of the experimental tests in this study.

## 3. Results

### 3.1. Microstructure and Compounds of As-Cast Alloy

**Figure 2:**
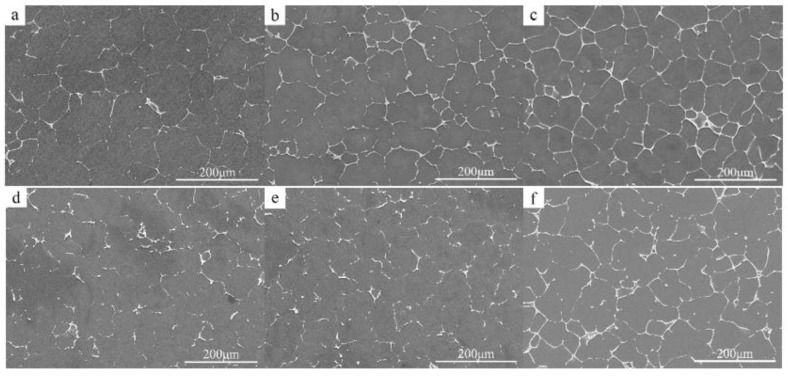
SEM microstructure images of the as-cast alloys. (**a**) ZCI, (**b**) ZCII, (**c**) ZCIII, (**d**) ZCIV, (**e**) ZCV, (**f**) ZCVI.

Figure 2 shows the SEM images of the as-cast microstructure of the experimental alloy, which can be seen to consist mainly of α-Mg matrix and a gray second phase on the grain boundaries. By increasing the content of Ce in the alloy alone, the density of the second phase at the grain boundaries increases significantly. It changes from intermittent distribution to continuous distribution, especially at the triangular grain boundaries, where the degree of aggregation is significantly increased, with typical eutectic organization characteristics. No obvious second phase was observed inside the grains. Changing the content of element Zn alone has a small effect on the amount and the distribution of the second phase, with only a small increase in the second phase in the 6 wt.% alloy.

**Figure 3:**
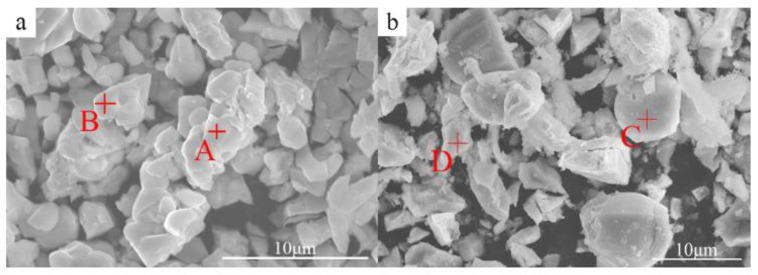
The morphologies of the secondary phases separated from experiment alloy: (**a**) ZCIII, (**b**) ZCVI.

Figure 3 shows the SEM photographs of the typical morphology of the solid phase isolated from the metallographic organization of ZCIII and ZCVI alloys. The compounds are mainly arranged in granular piles at the grain boundaries, showing a rounded appearance with mutual adhesion. The morphology of the solid phase isolates of the two experimental alloys is similar. The results of EDS analysis (as shown in Table 2) indicate that the main part of the compound is the Mg–Zn–Ce ternary compound, corresponding to points A and C in Figure 3. The content of Ce in the Mg–Zn–Ce ternary compound is basically the same, and the content of Zn increases with the increase of the addition, while the content of Mg in it decreases with the increase of Zn. This indicates that the binary replacement solid solution formed by Zn atoms in this compound can replace Mg at some positions. D. Kevorkov et al. [22] determined the chemical formula of this phase as (Mg, Zn)_12_Ce. A few Mg–Zn and Zn–Zr binary phases were detected in the fine bulk particles in the figure, corresponding to points B and D in Figure 3, respectively.

**Figure 4:**
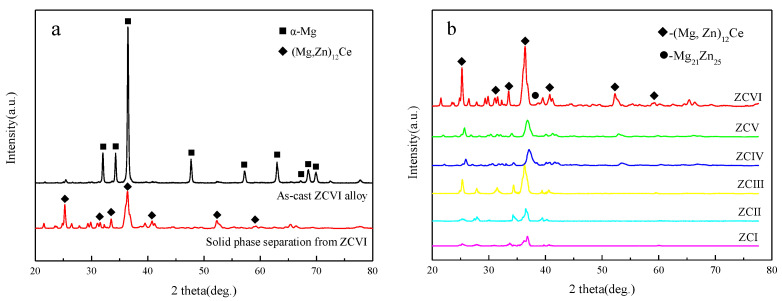
XRD results of as-cast alloy and its solid separation. (**a**) ZCVI, (**b**) Comparison of XRD results of alloy solid phase separation.

Figure 4 shows the XRD spectra of the cast alloy and its solid phase separated. The direct calibration of ZCVI alloy in Figure 4a resulted in an -Mg phase, and the second phase could not be accurately calibrated due to the low peak. After the phase separation, the -Mg phase diffraction peak disappears completely, and only the second phase particles remain in the solid phase separated. Due to the enrichment of the second phase, the calibration of each phase in the alloy solid-phase separates has high reliability. The calibration results in Figure 4b show that the solid phase separation includes the (Mg, Zn)_12_Ce phase and the Mg_21_Zn_25_ phase. The Mg–Zn and the Zn–Zr phases may be too small and entrapped in a large number of particles to be effectively calibrated. Based on the relative intensities of the (Mg, Zn)_12_Ce phase diffraction peaks, it can be seen that the proportion of the (Mg, Zn)_12_Ce phase increases with the increase of Ce and Zn content. The position of the main peak of the (Mg, Zn)_12_Ce phase in the figure shows a small shift, which is caused by the change of Zn content in the Mg–Zn–Ce ternary phase.

**Figure 5:**
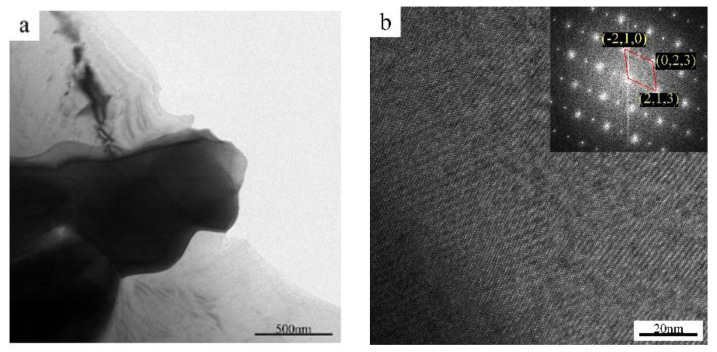
TEM micrograph of ZCVI alloy (**a**) Bright field TEM image, (**b**) High-resolution image and its FTT patterns.

The elements Mg, Zn, and Ce can form four major intermetallic compounds, including (Mg_1−x_Zn_x_)_12_Ce, (Mg_1−x_Zn_x_)_10_Ce, Mg_7_Zn_12_Ce, and Mg_3_Zn_5_Ce [13]. The selected area electron diffraction (SAED) pattern calibration results and high-resolution images of the prepared thin film specimens of ZCVI alloy are shown in Figure 5. The calibration results show that the distances of the center near diffraction spots are 4.844 Å, 4.915 Å, and 5.024 Å, and the angles are 59.35° and 61.14°, which are similar to the distances and the angles of the (−2, 1, 0), (0, 2, 3), and (2, 1, 3) crystal planes of the Mg_12_Ce phase, respectively. The TEM-EDS results show that the phases are all composed of Mg, Zn, and Ce. The atomic percentages of Mg, Zn, and Ce are 50.13%, 42.18%, and 7.7%, respectively, and the ratio of the sum of Mg and Zn atoms to Zn is 11.98:1. Therefore, the diffraction pattern calibration result is the (Mg, Zn)_12_Ce phase, which is consistent with the X-ray diffraction analysis result.

The Ce element in the alloy mainly exists as a solid solution and Mg–Zn–Ce ternary phases. Based on the determination of the rare earth compound phase as the (Mg, Zn)_12_Ce phase, the mass fraction of the (Mg, Zn)_12_Ce phase in each alloy can be calculated from the Ce element content (the difference between the Ce element content and the solid solution amount), assuming that the (Mg, Zn)_12_Ce phase follows the stoichiometric ratios determined by SEM-EDS and TEM-EDS, and the results are shown in Table 3.

### 3.2. Microstructure and Compounds of Homogeneous Alloy

**Figure 6:**
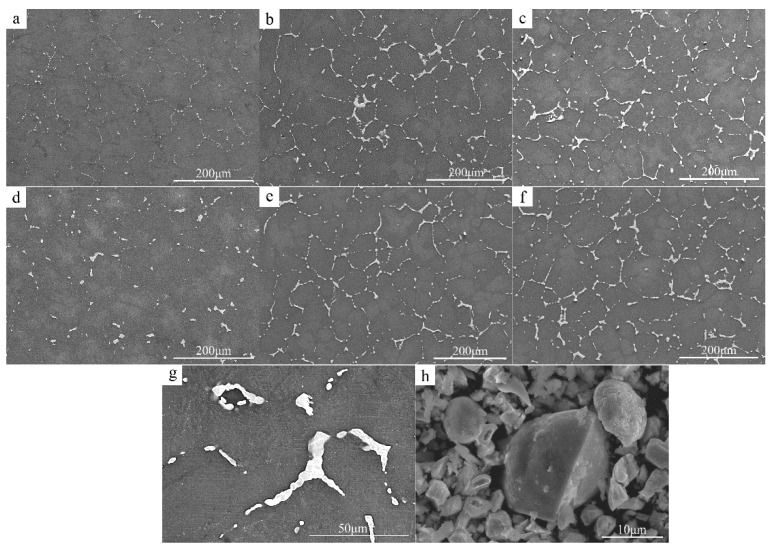
SEM microstructure images of the as-homogenized alloys. (**a**) ZCI, (**b**) ZCII, (**c**) ZCIII, (**d**) ZCIV, (**e**) ZCV, (**f**) ZCVI, (**g**) The morphology of ZCVI compound, (**h**) Separated solid phases sample of ZCⅥ.

Figure 6 shows the microstructure of the experimental alloy after the homogenization treatment. The second phase maintains the characteristics of interphase distribution similar to that of an as-cast state. However, at some locations, the second phase changed from continuous network distribution to intermittent distribution and concentrated at triangular grain boundaries and at other locations. Figure 6g shows the microstructure of ZCVI alloy in a homogeneous state. The outline of the compound is clearer and smoother than that in the as-cast state. The slender second phase between two adjacent grains is obviously reduced, while the second phase at the triangular grain boundary is increased. Figure 6h shows the separated solid phase morphology of a homogeneous ZCVI alloy, and larger second phase particles appear in the separated material.

**Figure 7:**
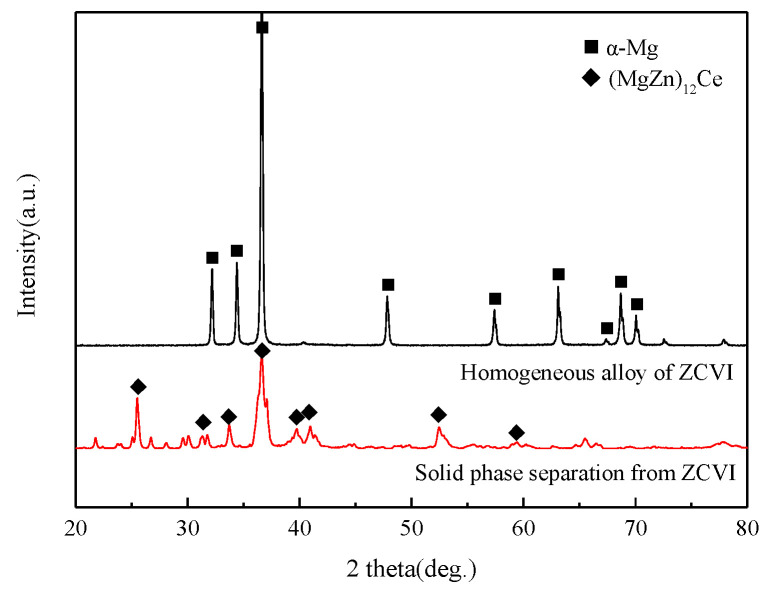
XRD spectrums of solid phases separated from homogeneous alloys.

Figure 7 is the XRD spectrum of a typical homogeneous alloy and separated solid phase. Only the (Mg, Zn)_12_Ce phase exists in the calibration results of the homogeneous alloy, which indicates that the (Mg, Zn)_12_Ce phase can exist stably at this homogenization temperature. As a result, the Mg–Zn phase was not calibrated, indicating that the non-equilibrium phase such as Mg–Zn was fully decomposed in the homogenization process. As only the (Mg, Zn)_12_Ce phase in the homogeneous alloy compound contains the Ce element, the mass fraction of the (Mg, Zn)_12_Ce phase in the alloy can be calculated according to the Ce content in the compound (the difference between the Ce addition amount and the solid solution amount), and the results are shown in Table 4.

**Figure 8:**
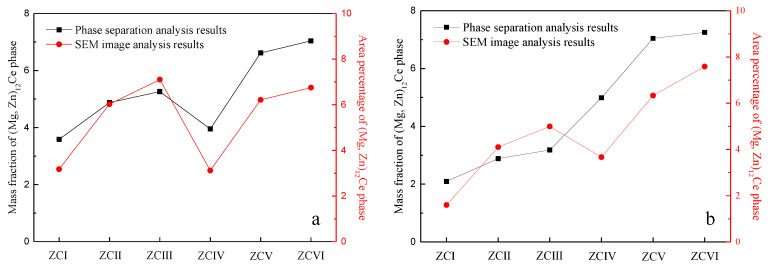
Comparison of quantitative analysis results of the (Mg, Zn)_12_Ce phase. (**a**) As-cast alloys, (**b**) homogeneous alloys.

To verify the accuracy of the quantitative analysis results of the (Mg, Zn)_12_Ce phase, SEM images of the as-cast and the homogenized states were analyzed in the study by using image analysis software, and the results are shown in Figure 8. The measured mass fraction of the (Mg, Zn)_12_Ce phase is shown on the left axis (black) and the area percentage of the (Mg, Zn)_12_Ce phase is shown on the right axis (red), which shows similar values and the same trend of (Mg, Zn)_12_Ce phase content with alloy composition.

### 3.3. Hot Deformation Microstructure and Mechanical Properties

**Figure 9:**
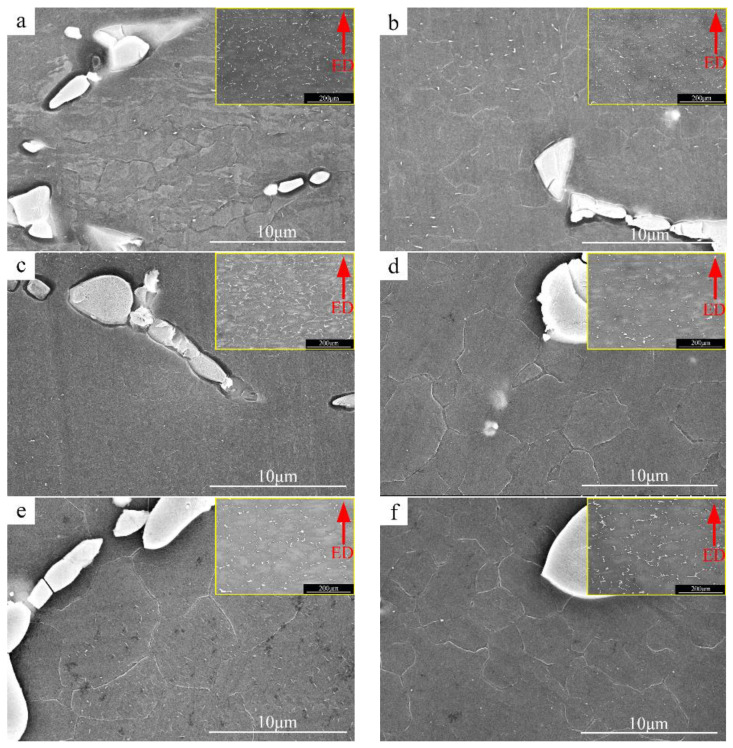
SEM microstructure image of deformed alloys. Red arrow direction is the extrusion direction (ED) (**a**) ZCI, (**b**) ZCII, (**c**) ZCIII0, (**d**) ZCIV, (**e**) ZCV, (**f**) ZCVI.

Figure 9 shows the SEM images of the heat deformation organization along the extrusion direction (ED), and its low magnification microstructures are shown in the upper right corner. The low-power SEM images show that the deformed microstructure includes the (Mg, Zn)_12_Ce phase distributed along the grain boundary and large extruded grains. In the high-power SEM image, besides the second phase broken during extrusion, a large number of dynamically recrystallized grains are distributed at the grain boundary, and the dynamically recrystallized grains are concentrated around the broken (Mg, Zn)_12_Ce phase. The above results show that a large number of initial grains remain during hot deformation, so that complete dynamic recrystallization is impossible, and a large number of dynamic recrystallization grains can be formed in the (Mg, Zn)_12_Ce phase.

**Figure 10:**
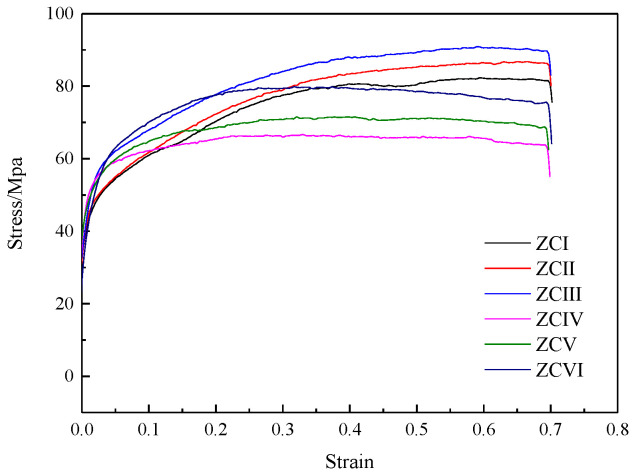
Stress–strain curve of experimental alloy.

The stress–strain curves of the experimental alloy are shown in Figure 10. The curve shows a fine serrated shape due to the joint effect of dynamic softening and work-hardening, which indicates that the softening and the hardening effects continue to interact and lead each other during the deformation process. When the strain is very small, the dislocations proliferate a lot, and the interaction between dislocations is enhanced, which becomes the resistance to their movement, and the process of hardening plays the main role at this time. When the strain continues to increase, the dynamic softening comes into play when the distortion energy caused by the dislocation stress field accumulates to a certain level. In the initial stage, the stress of ZCI, ZCII, and ZCII alloys increased rapidly, and work hardening played a dominant role. With the deformation process, the dynamic softening was enhanced and the hardening rate decreased, but the hardening still dominated. ZCIV, ZCV, and ZCVI alloys are similar to the above alloys before reaching their peak stress. However, after the peak stress continues to increase with the strain, the dynamic softening begins to dominate, and the stress gradually decreases. At the same time, the curve shows that the alloy with 6 wt.% Zn content has a stronger dynamic softening effect, but its strength is lower than that of the 3 wt.% alloy.

**Figure 11:**
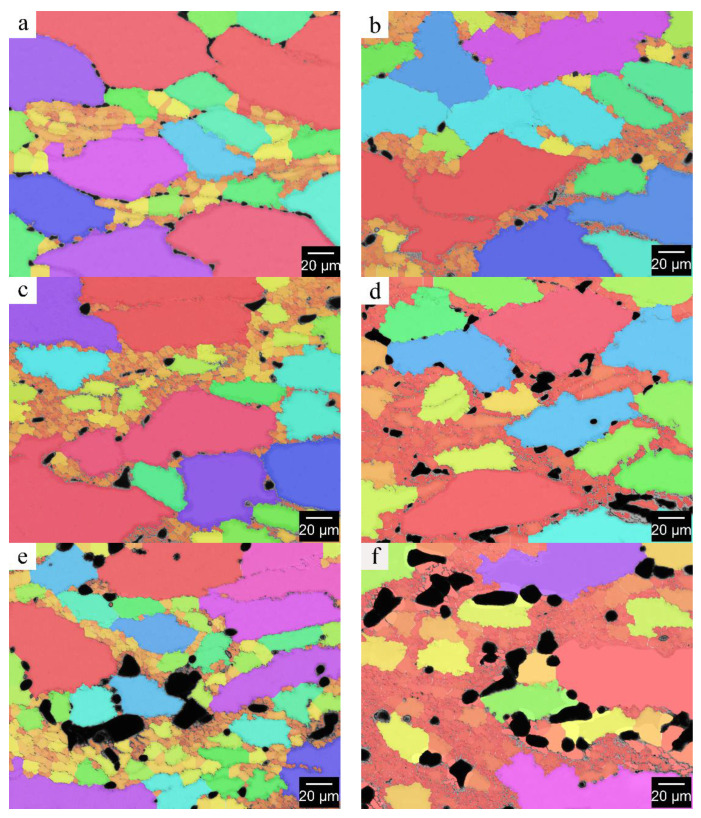
Recrystallization grain size and area calculation. (**a**) ZCI, (**b**) ZCII, (**c**) ZCIII, (**d**) ZCIV, (**e**) ZCV, (**f**) ZCVI.

In order to deeply investigate the microstructural characteristics of dynamic recrystallized grains during the thermal deformation of Mg-Zn-Ce-Zr alloy, the average size (μm), average area (μm^2^) and area ratio of recrystallized grains (shown in Table 5) were obtained by extracting the recrystallized grains with image analysis software (as shown in Figure 11). The results show that the change of Ce content and of Zn content can obviously affect the microstructure characteristics of recrystallized grains, and the increase of Ce or Zn content alone can reduce the size of dynamic recrystallized grains and the area ratio of recrystallized areas. The content of Ce and of Zn in ZCVI alloy is the highest, which has the most significant effect on dynamic recrystallization, and the recrystallization area ratio increases greatly.

## 4. Discussion

### 4.1. Effect of Ce and Zn Content on the Formation of the (Mg, Zn)_12_Ce Phase

The analysis of the microstructure of the as-cast alloy revealed that the number of rare earth compounds and their Zn and Ce contents did not follow the same proportional change when the addition of Zn and Ce elements increased proportionally, indicating that there are other factors affecting the content of elements involved in the formation of the compounds and that the initial element addition is not a direct factor affecting the number of compounds. In Figure 1, the (Mg, Zn)_12_Ce phases are all distributed at the grain boundaries, indicating that they are formed after the matrix phase, so the analysis of the solidification process of the alloy can help to study the compositional factors affecting the number of compounds.

**Figure 12:**
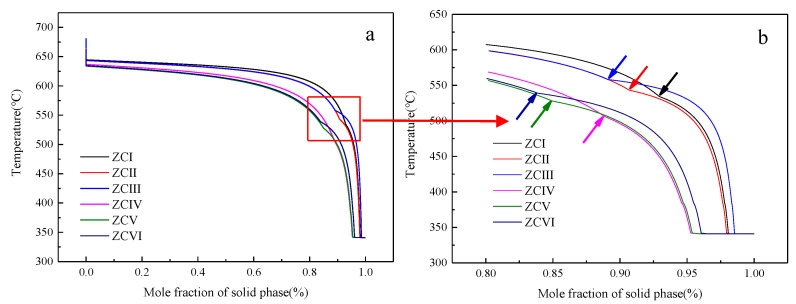
Scheil–Gulliver non-equilibrium solidification model. (**a**) Calculated results of S-G model for experimental alloy, (**b**) Enlarged view of the red area in the figure on the right.

The generalized phase diagram generally describes the condition that the system is in thermodynamic equilibrium. In fact, the diffusion coefficient of solute in the solid phase is very small, so equilibrium solidification is extremely difficult to achieve. The actual solidification process is usually closer to the Scheil–Gulliver cooling condition, that is, there is no diffusion in the solid phase, the liquid phase is uniformly mixed, and the solid–liquid interface is in a local equilibrium state [21,22]. The Scheil–Gulliver model calculation of the solidification process of Mg–Zn–Ce–Zr alloy is shown in Figure 12. During the cooling process, the -Mg matrix phase is first formed, and the position marked by the arrow in Figure 12b is the starting point of the precipitation of other phases in the remaining liquid phase. The Mg–Zn–Ce compound is formed in the remaining liquid phase after the solidification of the α-Mg phase. It can be seen that Zn and Ce elements can be divided into two parts: one part forms an -Mg solid solution, and the other part exists in the remaining melt. As the elements in the residual liquid phase directly participate in the formation process of the (Mg, Zn)_12_Ce phase, it is more accurate to describe the formation of the (Mg, Zn)_12_Ce phase by using the alloy components in the residual liquid phase.

**Figure 13:**
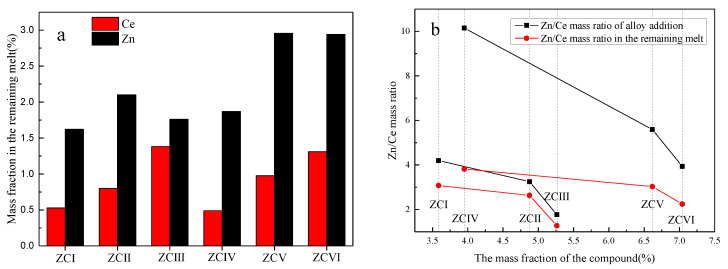
Mass fraction of elements in the remaining melt. (**a**) Calculated mass fraction of Ce and Zn elements in the remaining melt, (**b**) Correspondence between the Zn/Ce mass ratio and the compound mass fraction in the alloy and the remaining melt.

The Zn and Ce contents in the remaining melt are shown in Figure 13. With the increase of the Ce element, the Ce content in the remaining melt increases linearly, and it is not affected by the Zn content. The content of Zn in the remaining melt is mainly determined by the added amount. Figure 13b shows the relationship between the mass ratio of Zn/Ce in the alloy and the mass ratio of Zn/Ce in the remaining melt and the number of compounds. The lower Zn/Ce mass ratio is beneficial to the formation of the (Mg, Zn)_12_Ce phase, and the change trend of compounds is similar, while the number of compounds formed in the alloy with 6 wt.% Zn content is obviously greater. It shows that the conditions for increasing the number of compounds are increasing the content of Zn and Ce in the remaining melt and decreasing the mass ratio of Zn/Ce.

### 4.2. Effect of the (Mg, Zn)_12_Ce Phase Content on Dynamic Recrystallization

**Figure 14:**
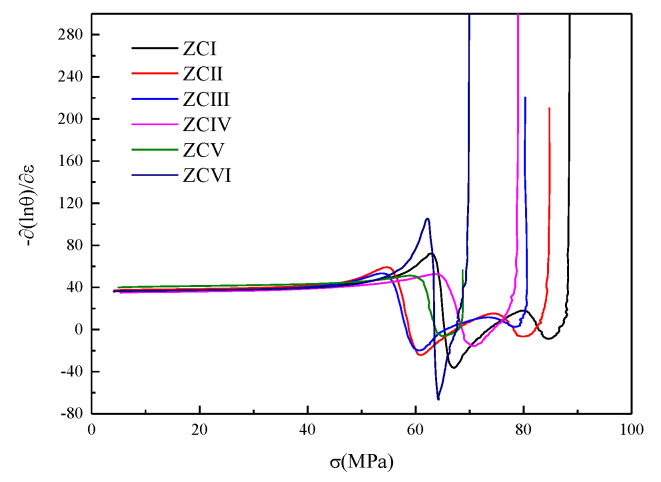
Curves of $-\left(\frac{\partial \ln\theta }{\partial \varepsilon }\right)-$*σ*.

The critical condition of dynamic recrystallization (DRX) is an important parameter to study the evolution of dynamic recrystallization, and the work hardening rate method is often used to judge the condition of dynamic recrystallization of magnesium alloys [23]. The critical condition for the softening behavior of the material DRX can be proved as $\frac{\partial }{\partial \left(\sigma \right)}\left(-\frac{\partial \theta }{\partial \sigma }\right)=0$ (i.e., the inflection point of the −∂(lnθ)/∂ε curve) by using the principle of incremental work balance of the thermodynamic system. Figure 14 shows the results of the partial derivative calculation of the work-hardening rate curve. The critical conditions for dynamic recrystallization of the experimental alloy can be determined from the minimal values of the curves. Table 6 displays the data for the minima. As the number of (Mg, Zn)_12_Ce phases increases, the critical stress for dynamic recrystallization decreases and the corresponding critical strain decreases, indicating that the alloy is more likely to start dynamic recrystallization during thermal deformation.

**Figure 15:**
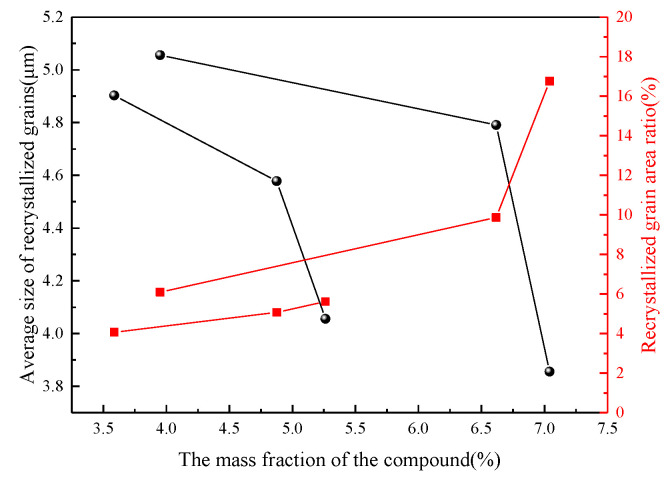
Influence of the number fraction of rare earth compounds on recrystallized grains.

The second phase during thermal deformation can act as a nucleation point for recrystallized particles, i.e., play a particle stimulated nucleation (PSN) mechanism, and what role the particles play depends on the size, spacing, and fraction of the particles [24]. The intermittently distributed second phase after the homogenization treatment (shown in Figure 5) and the broken second phase particles after thermal deformation (shown in Figure 7) all indicate that the conditions for the PSN mechanism to function are satisfied in the alloy. During the heat deformation processing, strain gradient regions are generated in these rare earth compounds with certain dimensions. These regions have a high dislocation density; a large orientation; and, around the grains, subgrain boundaries move rapidly to form new high-angle grain boundaries [25]. All of these locations are effective nucleation points for recrystallized grains. In addition, clustering of second-phase particles on triangular grain boundaries appears in the microstructure after the homogenization treatment. It has been shown that clustered second-phase particles are equally beneficial in promoting the DRX behavior during extrusion [26]. The results in Figure 15 show that the increase in the number of (Mg, Zn)_12_Ce phases reduces the size of the recrystallized grains, while increasing the area fraction of recrystallized grains in the alloy, which is consistent with the results in previous reports.

### 4.3. Effect of (Mg, Zn)_12_Ce Phase Content on Mechanical Properties

**Figure 16:**
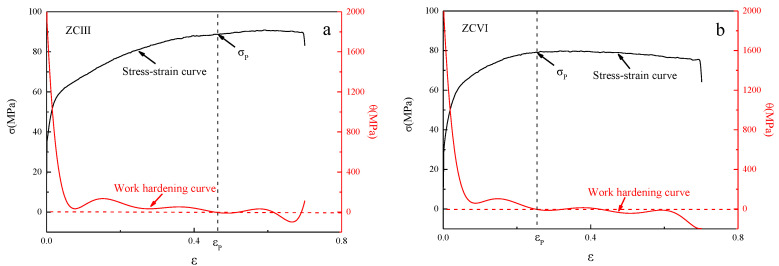
Typical stress–strain and *θ*−*ε* curves. (**a**) ZCIII alloy, (**b**) ZCVI alloy.

Figure 16 shows the stress–strain curves and the work hardening curves of two typical experimental alloys. When the strain is $\varepsilon_{p}$, the alloy reaches the peak stress $\sigma_{p}$, and the *θ* value in the work hardening curve of the alloy decreases to 0. In the flow stress curve of ZCⅢ alloy, the stress tends to be stable after reaching the peak stress, and it does not show typical DRX softening characteristics. At this time, the dynamic softening mechanism is dominated by the dynamic recovery mechanism (DRV). However, during the subsequent strain increase, the softening effect of DRX gradually increases, and DRX grains appear at the grain boundaries of the alloy. The flow stress curve of ZCIV alloy shows obvious DRX softening characteristics. The flow stress gradually decreases after reaching the peak stress, and the dynamic softening mechanism is dominated by DRX. The above analysis shows that the content of the Zn element will affect the dynamic softening mechanism of the alloy. The dynamic softening mechanism of low Zn alloy is mainly DRV, while that of high Zn alloy is mainly DRX, and the effect of DRX is stronger in the alloy with a high content of the (Mg, Zn)_12_Ce phase.

**Figure 17:**
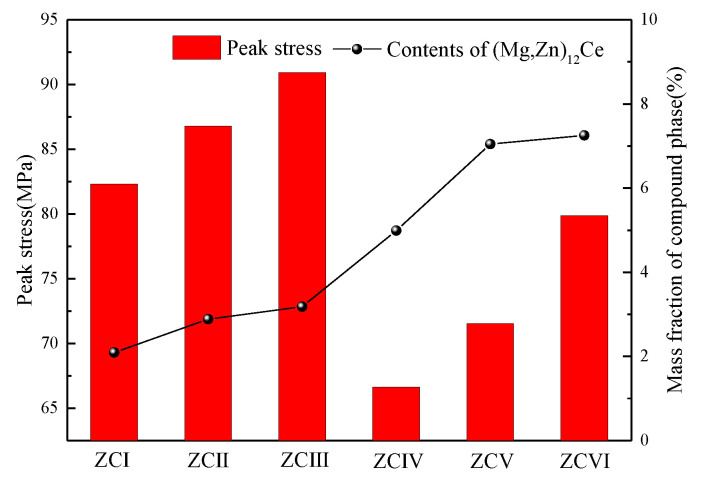
Peak stress of experimental alloys.

Figure 17 shows the relationship between the peak stress and the number of (Mg, Zn)_12_Ce phases in the experimental alloy. With the increase of compound content in alloys with different Zn content, the peak stress increases, and the changing trend is consistent. However, it can be seen that the peak stress is obviously lower in 6 wt.% Zn alloy with a higher number of compounds, which is attributed to the role of the (Mg, Zn)_12_Ce phase in the process of thermal deformation. The increase in the number of (Mg, Zn)_12_Ce phases reduces the critical conditions for dynamic recrystallization and greatly advances the dynamic softening effect in the alloy. In the corresponding stress–strain curves, ZCIV, ZCV, and ZCVI alloys show peak stress at small strain. In addition, the increase in the number of (Mg, Zn)_12_Ce phases also strengthens the PSN mechanism, provides conditions for the nucleation of recrystallized particles, and inhibits their growth, which corresponds to the stress reduction stage in the stress–strain curve. The above analysis results show that controlling the amount of the (Mg, Zn)_12_Ce phase can not only predict the properties of the alloy during the current deformation process but also adjust the trend of the stress–strain curve to obtain ideal mechanical properties.

## 5. Conclusions

In this study, the mass fraction of the (Mg, Zn)_12_Ce phase in the alloy was obtained based on a chemical phase separation method. The effects of Zn and of Ce contents in the remaining liquid phase on the (Mg, Zn)_12_Ce phase content were analyzed in combination with the quantitative analysis results, revealing the effects of the (Mg, Zn)_12_Ce phase content on the mechanical properties, and the main conclusions can be summarized as follows.

The Mg–Zn–Ce compound in the as-cast alloy is only the (Mg, Zn)_12_Ce phase, and the Zn and Ce content will affect the amount of the compound formed in the alloy. With the increase of Zn and Ce content, the (Mg, Zn)_12_Ce content increased from the lowest 3.586 wt.% to the highest 7.040 wt.%. Additionally, the (Mg, Zn)_12_Ce phase can be stable in the homogenization treatment due to its higher thermal stability.

The (Mg, Zn)_12_Ce phase is formed in the liquid phase remaining after the solidification of the α-Mg phase is completed. Therefore, the formation of the (Mg, Zn)_12_Ce phase was more accurately analyzed using the content of each element in the remaining liquid phase. The maximum compound content for different Zn contents can be obtained by controlling the Zn/Ce mass ratio in the remaining melt to below 2.5 in the composition designed for the study.

The increase in the amount of the (Mg, Zn)_12_Ce phase can reduce the critical strain and the critical stress of dynamic recrystallization and promote the dynamic recrystallization process together with the PSN nucleation mechanism of the (Mg, Zn)_12_Ce phase. In addition, the work-hardening curves indicate that alloys with 6 wt.% Zn elements tend to undergo a DRX mechanism, while alloys with 3 wt.% Zn elements tend to undergo a DRV mechanism.

At present, we have studied Mg–Zn–Zr alloys with different types of rare earths added using chemical phase separation methods, all of which can achieve the expected results. However, this method is currently limited to Mg–Zn-based magnesium alloys and a few Mg-Al-based magnesium alloys. Due to differences in the physical and the chemical properties of the elements in the alloys, the adjustment of the reagents used in the separation solution can expand the application area of the phase separation method.

## Figures and Tables

**Table 1:** Design composition and measured composition of Mg–Zn–Ce–Zr alloys.

Alloy Code	Nominal Alloys	Composition (wt.%)			
		Zn	Ce	Zr	Mg
ZCI	Mg–3Zn–0.5Ce–0.5Zr	2.599	0.619	0.517	Bal.
ZCII	Mg–3Zn–1.0Ce–0.5Zr	3.042	0.936	0.522	Bal.
ZCIII	Mg–3Zn–1.5Ce–0.5Zr	2.697	1.525	0.513	Bal.
ZCⅣ	Mg–6Zn–0.5Ce–0.5Zr	5.410	0.533	0.570	Bal.
ZCⅤ	Mg–6Zn–1.0Ce–0.5Zr	5.900	1.055	0.510	Bal.
ZCⅥ	Mg–6Zn–1.5Ce–0.5Zr	5.560	1.412	0.540	Bal.

**Table 2:** EDS Analysis Results of Typical Alloy Solid Phase Separation.

Mark Position	Composition (at.%)			
	Zn	Ce	Zr	Mg
A	17.93	7.21		74.86
B	71.80		28.20	
C	22.73	6.95		70.32
D	59.46			40.54

**Table 3:** The mass fraction of the (Mg, Zn)_12_Ce phase in the as-cast alloys.

	ZCI	ZCII	ZCIII	ZCIV	ZCV	ZCVI
(Mg, Zn)_12_Ce phase	3.586	4.875	5.262	3.950	6.617	7.040

**Table 4:** The mass fraction of the (Mg, Zn)_12_Ce phase in the homogeneous alloys.

Compound	ZCI	ZCII	ZCIII	ZCIV	ZCV	ZCVI
(Mg, Zn)_12_Ce phase	2.093	2.884	3.183	4.990	7.045	7.253

**Table 5:** Measurement results of recrystallized grains.

	ZCI	ZCII	ZCIII	ZCIV	ZCV	ZCVI
Average diameter (μm)	4.703	4.578	4.056	5.056	4.791	3.856
Average area (μm^2^)	6.885	5.706	5.593	8.344	6.866	5.608
Area fraction (%)	4.068	5.073	5.616	6.098	9.868	16.760

**Table 6:** Critical strain in experimental alloys of various compositions.

	ZCI	ZCII	ZCIII	ZCIV	ZCV	ZCVI
$\varepsilon_{c}$	0.09693	0.09307	0.09120	0.15520	0.10496	0.09206
$\sigma_{c}$	67.089	60.982	60.636	70.596	64.278	62.183

## Data Availability

All data are available upon request. The data presented in this study are available on request from the corresponding author.
